# Radiomics analysis of contrast-enhanced computed tomography in predicting the International Neuroblastoma Pathology Classification in neuroblastoma

**DOI:** 10.1186/s13244-023-01418-5

**Published:** 2023-06-14

**Authors:** Haoru Wang, Mingye Xie, Xin Chen, Jin Zhu, Li Zhang, Hao Ding, Zhengxia Pan, Ling He

**Affiliations:** 1 Department of Radiology, Children’s Hospital of Chongqing Medical University, National Clinical Research Center for Child Health and Disorders, Ministry of Education Key Laboratory of Child Development and Disorders, Chongqing Key Laboratory of Pediatrics, No. 136 Zhongshan Road 2, Yuzhong District, Chongqing, 400014 China; 2 Department of Pathology, Children’s Hospital of Chongqing Medical University, National Clinical Research Center for Child Health and Disorders, Ministry of Education Key Laboratory of Child Development and Disorders, Chongqing Key Laboratory of Pediatrics, No. 136 Zhongshan Road 2, Yuzhong District, Chongqing, 400014 China; 3 Department of Cardiothoracic Surgery, Children’s Hospital of Chongqing Medical University, National Clinical Research Center for Child Health and Disorders, Ministry of Education Key Laboratory of Child Development and Disorders, Chongqing Key Laboratory of Pediatrics, No. 136 Zhongshan Road 2, Yuzhong District, Chongqing, 400014 China

**Keywords:** Neuroblastoma, Pathology, Radiomics, Computed tomography

## Abstract

**Purpose:**

To predict the International Neuroblastoma Pathology Classification (INPC) in neuroblastoma using a computed tomography (CT)-based radiomics approach.

**Methods:**

We enrolled 297 patients with neuroblastoma retrospectively and divided them into a training group (*n* = 208) and a testing group (*n* = 89). To balance the classes in the training group, a Synthetic Minority Over-sampling Technique was applied. A logistic regression radiomics model based on the radiomics features after dimensionality reduction was then constructed and validated in both the training and testing groups. To evaluate the diagnostic performance of the radiomics model, the receiver operating characteristic curve and calibration curve were utilized. Moreover, the decision curve analysis to assess the net benefits of the radiomics model at different high-risk thresholds was employed.

**Results:**

Seventeen radiomics features were used to construct radiomics model. In the training group, radiomics model achieved an area under the curve (AUC), accuracy, sensitivity, and specificity of 0.851 (95% confidence interval (CI) 0.805–0.897), 0.770, 0.694, and 0.847, respectively. In the testing group, radiomics model achieved an AUC, accuracy, sensitivity, and specificity of 0.816 (95% CI 0.725–0.906), 0.787, 0.793, and 0.778, respectively. The calibration curve indicated that the radiomics model was well fitted in both the training and testing groups (*p* > 0.05). Decision curve analysis further confirmed that the radiomics model performed well at different high-risk thresholds.

**Conclusion:**

Radiomics analysis of contrast-enhanced CT demonstrates favorable diagnostic capabilities in distinguishing the INPC subgroups of neuroblastoma.

**Graphical Abstract:**

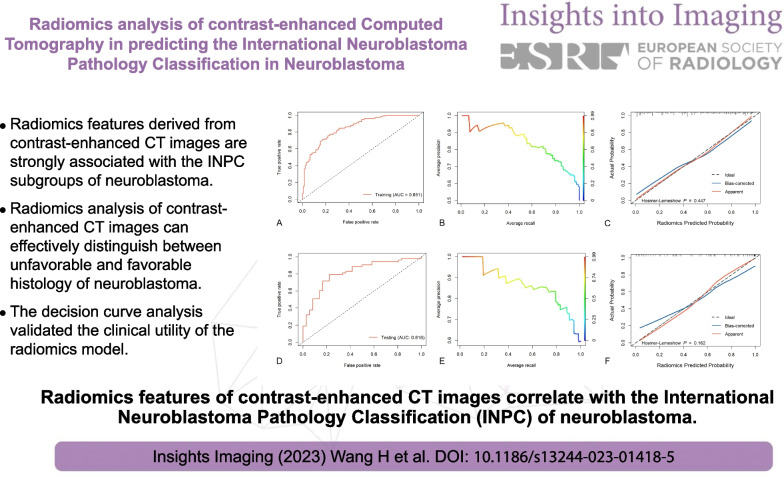

**Critical relevance statement:**

Radiomics features of contrast-enhanced CT images correlate with the International Neuroblastoma Pathology Classification (INPC) of neuroblastoma.

**Supplementary Information:**

The online version contains supplementary material available at 10.1186/s13244-023-01418-5.

## Key points

- Radiomics features derived from contrast-enhanced CT images are strongly associated with the INPC subgroups of neuroblastoma.
- Radiomics analysis of contrast-enhanced CT images can effectively distinguish between unfavorable and favorable histology of neuroblastoma.
- The decision curve analysis has validated the clinical utility of the radiomics model.

**Supplementary Information:**

The online version contains supplementary material available at 10.1186/s13244-023-01418-5.

## Introduction

Neuroblastoma is a malignant solid tumor in children that accounts for 15% of cancer-related deaths in children [1]. The International Neuroblastoma Pathology Classification (INPC) is an important classification system for neuroblastoma, providing a reference for different treatment stratification based on various risk factors associated with poor prognosis [2]. INPC classifies neuroblastoma into two subgroups based on patient age, tumor histological subtype, differentiation grade, and mitosis karyorrhexis index (MKI): favorable histology (FH) and unfavorable histology (UFH) [3]. INPC is highly prognostic, with significant differences in predicted survival between FH and UFH, with a 3-year event-free survival rate that is much higher in FH than in UFH [4]. Age has long been considered the most important prognostic factor for neuroblastoma, while INPC could provide additional valuable prognostic information [5]. In addition to being linked to the genomic signature of neuroblastoma, INPC is also an essential element of the Children's Oncology Group neuroblastoma risk classification system [6, 7]. Therefore, INPC is essential for developing an effective stratified treatment strategy for neuroblastoma.

However, the assessment of INPC is complex and subjective, and the analysis of the same patient by multiple pathologists may lead to inconsistent results. Moreover, the pathological heterogeneity of neuroblastoma can cause different differentiation grades at different parts of the same tumor, thus reducing the accurate evaluation of INPC [8, 9]. Besides, the evaluation of MKI, an indicator of INPC, involves manual counting of 5000 cells under a microscope to determine the total number of cells undergoing karyorrhexis or in mitosis, which is a lengthy and laborious process [10]. Despite the significant success achieved through computer techniques in image analysis, determining MKI has proven to be a challenge [11]. Consequently, creating an automated and objective method to aid in the pathological classification of neuroblastoma is of great clinical interest.

Radiomics is a rapidly developing technology that combines big data and artificial intelligence to aid in diagnosis. By further analyzing medical images, it extracts quantitative features to reflect lesion heterogeneity and has demonstrated great potential in tumor staging, pathological subtyping, and prognosis prediction [12–14]. Thus, medical images can provide not only visual information about the disease, but also act as a digital representation of a pathological section. Liu et al. [15] demonstrated the potential of radiomics in neuroblastoma by incorporating it into machine learning models to predict outcomes. Other studies have also revealed that radiomics can be used to identify the pathological subtypes and genetic aberrations of neuroblastoma [16, 17]. Nevertheless, there has been only one report on the use of ^18^F-FDG PET/CT-based radiomics to predict INPC subgroups in neuroblastoma [18]. Therefore, the aim of this study was to predict the INPC subgroups in neuroblastoma using a CT-based radiomics approach.

## Materials and methods

### Study population

**Fig. 1:**
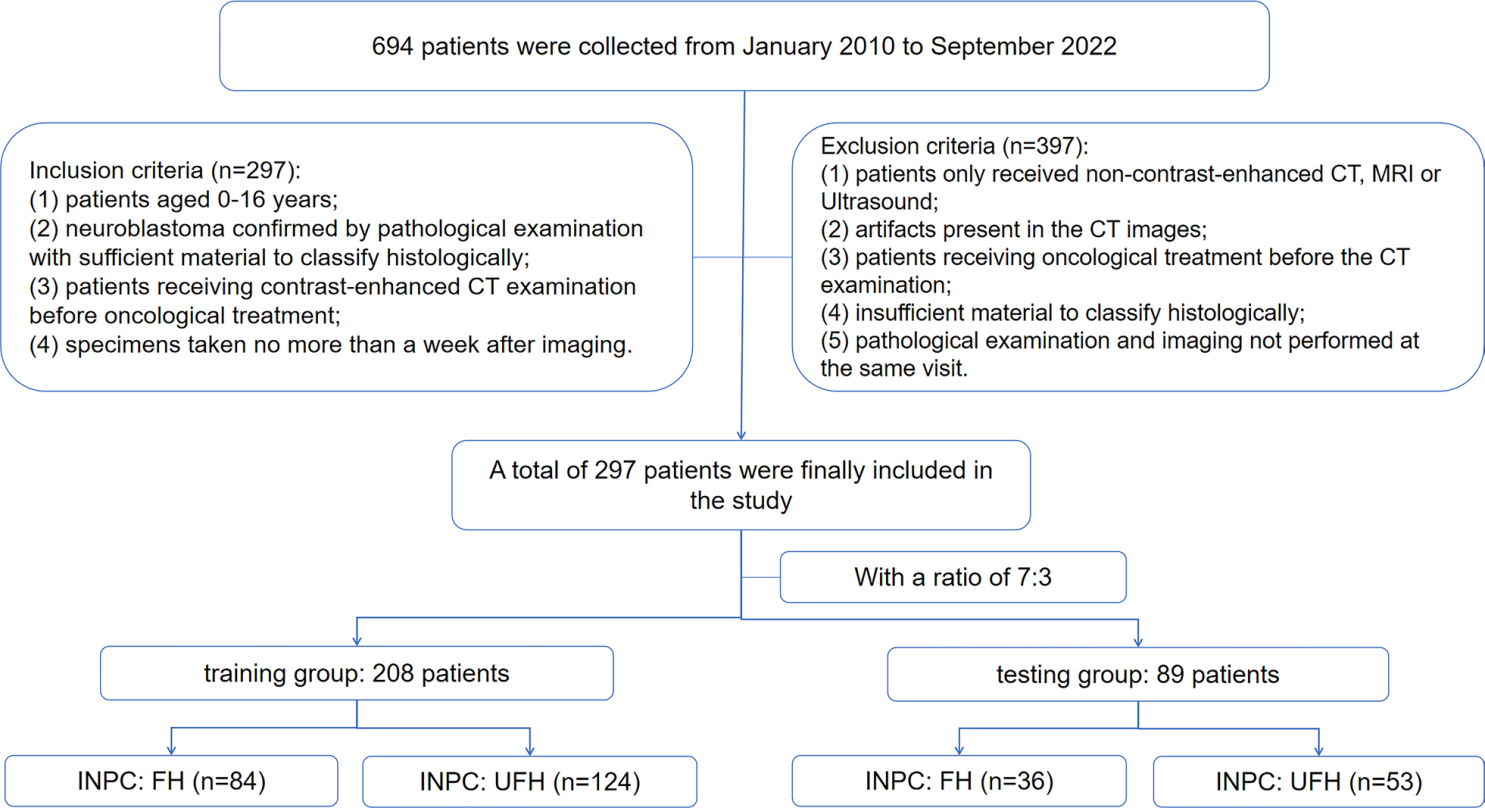
Patient selection pathway. INPC, International Neuroblastoma Pathology Classification; FH, favorable histology; UFH, unfavorable histology

This study was approved by the Ethics Committee of the Children’s Hospital of Chongqing Medical University, and the consent was waived due to its retrospective nature. The clinical and pathological data of children with pathologically confirmed neuroblastoma from our hospital between January 2010 and September 2022 were retrospectively collected. To divide the cohort into a training group and a testing group, a 7:3 stratified sampling was utilized. The inclusion criteria for this study were as follows: (1) patients aged 0–16 years; (2) neuroblastoma confirmed by pathological examination with sufficient material to classify histologically; (3) patients receiving contrast-enhanced CT examination before oncological treatment; (4) specimens taken no later than a week after imaging. The exclusion criteria were as follows: (1) patients only receiving non-contrast-enhanced CT, MRI, or ultrasound; (2) artifacts present in the CT images; (3) patients receiving oncological treatment before the CT examination; (4) insufficient material to classify histologically; (5) pathological examination and imaging not performed at the same visit. As shown in Fig. 1, the patient selection pathway was followed.

### INPC evaluation

Two pathologists were involved in the pathological analysis and any disagreements were resolved through discussion where the tumor was pathologically analyzed depended on the sampling method. If upfront complete resection was performed, the whole tumor was analyzed. If upfront complete resection was not performed, a biopsy or incomplete resection was conducted. Patients were divided into FH and UFH based on the histological subtype, differentiation degree, MKI and patient age [3, 19]. The definitions of FH and UFH can be found in Additional file 1: Fig. S1.

### CT acquisition

All patients underwent CT scans using a Lightspeed VCT (GE Healthcare) or Brilliance iCT (PHILIPS). The scanning parameters were set to a tube voltage of 80–120 kV, tube current of automatic, noise index of 12, scanning layer thickness of 5 mm, pitch of 0.6–1.1. An isotonic iodine contrast agent (Visipaque 320 mg I/mL, GE Healthcare) was used for the contrast agent protocol. The injection dose (mL) was calculated using the formula of 2 × body weight (kg), with a maximum dose of 80 mL. The contrast agent was injected from the peripheral superficial vein by a high-pressure syringe at a rate of 0.5–3.5 mL/s for 18–20 s, followed by flushing of the tube with saline at the same rate for 6–8 s. Initially, a CT scan without the use of a contrast agent was conducted. Subsequently, images in the arterial and venous phases were obtained at 20–28 s and 55–66 s after the injection of the contrast agent, respectively.

### Tumor segmentation and radiomics feature extraction

**Fig. 2:**
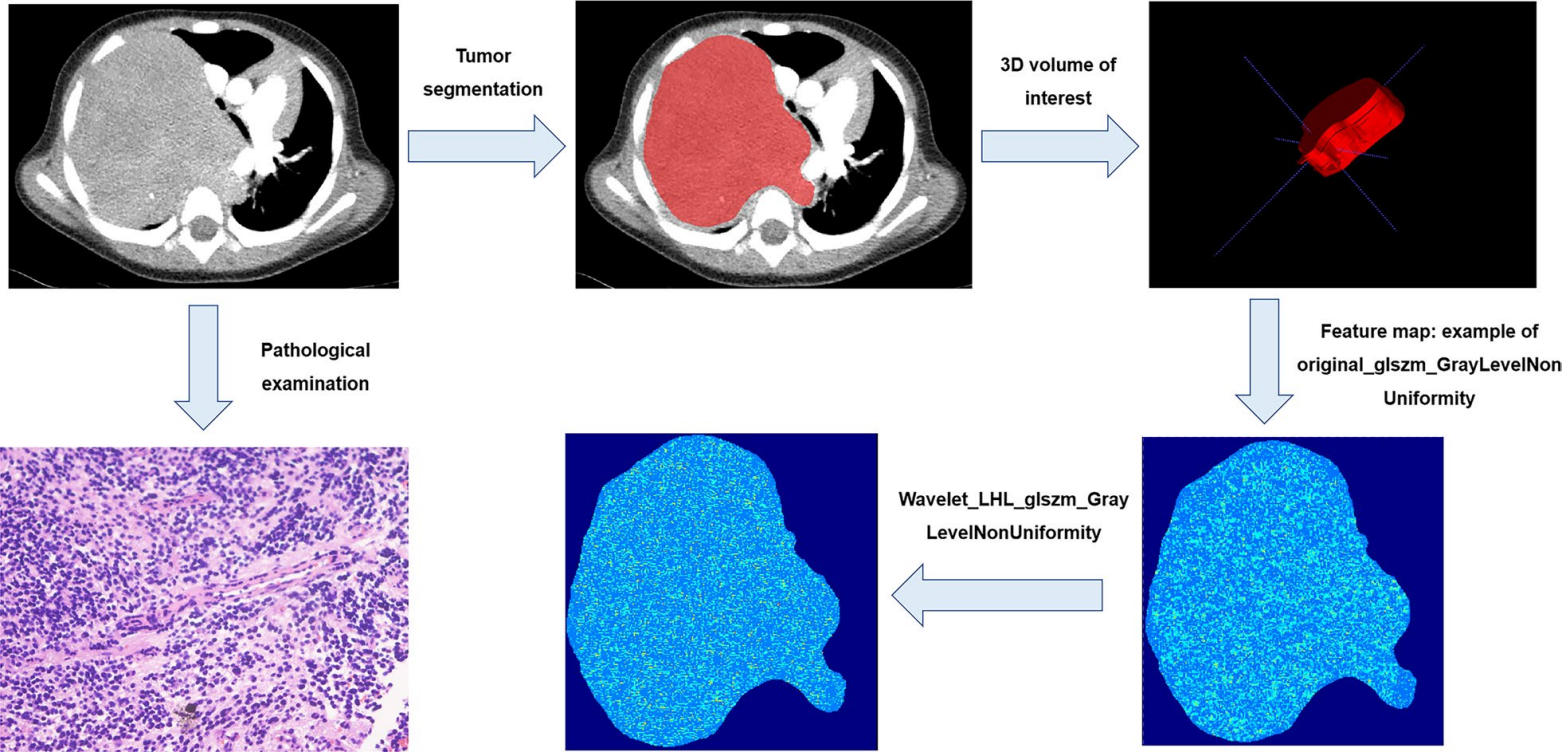
Example of tumor segmentation. The patient, an 8-year-old male, with thoracic poorly differentiated neuroblastoma, was classified as favorable histology

The contrast-enhanced CT images at the arterial phase were uploaded to ITK-SNAP (version 3.6.0) software for tumor segmentation. A radiologist with 3 years of experience, who was blinded to the pathological results, manually delineated the maximum five slices of the tumor area. The segmentation was subsequently verified by another radiologist with 14 years of experience. We segmented the area of interest to exclude vascular structures and organs that were encased, resulting in only the tumor region being included for radiomics feature extraction. An example of tumor segmentation can be seen in Fig. 2. To ensure generalization performance, voxel resampling (1 × 1 × 1 mm^3^) was conducted on CT images prior to radiomics features extraction. A total of 1046 radiomics features, including morphological features, histogram features and texture features, were extracted (Supplementary Table 1). The original features were transformed with Laplacian of Gaussian (LOG) and wavelet filters to generate higher-order features. To assess the dependability of radiomics features, a subset of 40 patients from the cohort underwent tumor re-segmentation and subsequent extraction of radiomics features.

**Table 1:** Demographic characteristics of the patients

Characteristics	FH (*n* = 120)	UFH (*n* = 177)	*p* value
Age (months)	12.5 (3.25, 37.75)	37.0 (22.0, 57.0)	< 0.001
Gender			0.659
Male	64 (53.3%)	99 (55.9%)	
Female	56 (46.7%)	78 (44.1%)	
Location			NA
Neck	1 (0.8%)	1 (0.6%)	
Neck-thorax	2 (1.7%)	0 (0.0%)	
Thorax	14 (11.7%)	12 (6.8%)	
Thorax-abdomen	2 (1.7%)	4 (2.2%)	
Abdomen	85 (70.8%)	152 (85.9%)	
Abdomen-pelvis	7 (5.8%)	6 (3.4%)	
Pelvis	9 (7.5%)	2 (1.1%)	
INRG stage			< 0.001
L1	25 (20.8%)	17 (9.6%)	
L2	71 (59.2%)	42 (23.7%)	
M	15 (12.5%)	115 (65.0%)	
MS	9 (7.5%)	3 (1.7%)	
Histological subtype			< 0.001
Poorly differentiated neuroblastoma	73 (60.8%)	132 (74.6%)	
Undifferentiated neuroblastoma	0 (0.0%)	20 (11.3%)	
Differentiated neuroblastoma	3 (2.5%)	2 (1.1%)	
Intermixed ganglioneuroblastoma	40 (33.3%)	0 (0.0%)	
Nodular ganglioneuroblastoma	4 (3.3%)	23 (13.0%)	

*FH* favorable histology, *UFH* unfavorable histology, *NA* not applicable, *INRG* international neuroblastoma risk group

### Radiomics feature selection

**Fig. 3:**
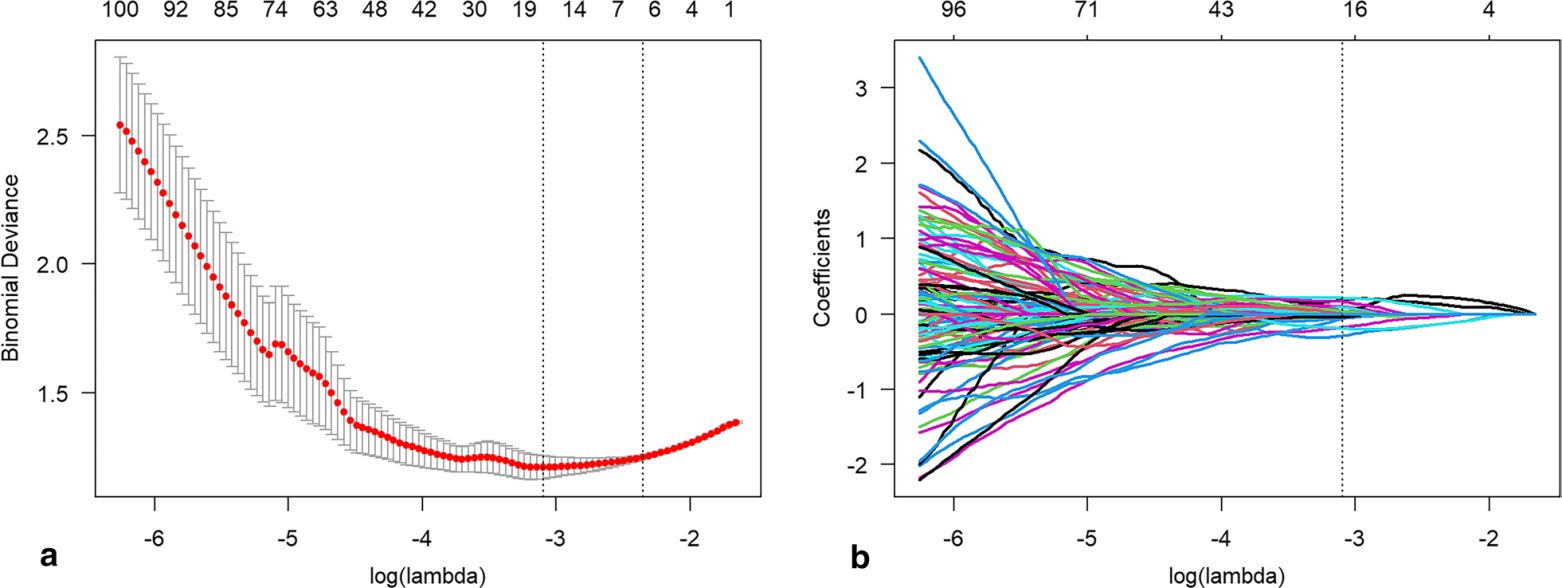
Pathway of feature selection using a least absolute shrinkage and selection operator algorithm. Figure **a** shows the selection of the optimal lambda value with minimum prediction error using a fivefold cross-validation method. Figure **b** shows the selection of the radiomics features with nonzero compression coefficients under the optimal lambda value

According to the radiomics features extracted from the twice tumor segmentation, the intra-class correlation coefficient (ICC) was calculated, and radiomics features with an ICC greater than 0.80 were selected. To increase the size of the minority class in the training group to match that of the majority class, a Synthetic Minority Over-sampling Technique (SMOTE) was employed before feature dimensionality reduction, with the k-neighbors set to 5. Pearson correlation coefficient (PCC) between the pairwise radiomics features was calculated, with the threshold set to 0.99. To select radiomics features, a least absolute shrinkage and selection operator (LASSO) algorithm was employed in conjunction with fivefold cross-validation, which was used to identify the best lambda value with the lowest prediction error. The radiomics features with nonzero compression coefficients were then selected based on the best lambda value, as illustrated in Fig. 3.

### Development and validation of the radiomics model

The logistic regression radiomics model was constructed using the final selected radiomics features and then validated in the training and testing groups. The calibration curve and Hosmer–Lemeshow goodness-of-fit test were employed to assess the consistency between the predicted and actual probabilities of the radiomics model in the training and testing groups. The *p* value was used to determine if the predicted probabilities of the radiomics model were significantly different from the actual probabilities, with a *p* value less than 0.05 indicating poor diagnostic performance. To further evaluate the clinical utility of the radiomics model, decision curve analysis was employed to analyze the net benefits of the radiomics model at different high-risk thresholds.

### Statistical analysis

Statistical analysis was performed using RStudio (version 4.1.1) and FeAture Explorer software (version 0.5.3) software [20]. Categorical data were compared between two groups using a chi-square test, and measurement data between two groups were compared using Student t test or Mann–Whitney U test. The diagnostic performance of radiomics model was evaluated using receiver operating characteristic (ROC) curves and precision-recall (PR) curves, with the area under the curve (AUC), 95% confidence interval (CI), accuracy, sensitivity, specificity, negative prediction value (NPV) and positive prediction value (PPV) used to assess the performance. Statistical significance was determined when the *p* value was less than 0.05.

## Result

### Demographic data

This study included a total of 297 cases, with 163 males and 134 females. The median age of the patients was 30.0 months, with a range of 0.07–161 months. There were 120 cases in FH group and 177 cases in UFH group. In the FH group, there were 64 males and 56 females, with a median age of 12.5 months and an age range of 0.07–150 months. In the UFH group, there were 99 males and 78 females, with a median age of 37.0 months and an age range of 1–161 months. The age and international neuroblastoma risk group (INRG) stage were found to be statistically different between FH and UFH (*p* < 0.001), but gender was not statistically different between the two groups. Table 1 presents the demographic data of the FH and UFH groups, while Table 2 shows that there was no statistically significant difference in demographic data between the training and testing groups.

**Table 2:** Comparison of demographic characteristics of the patients between the training group and testing group

Characteristics	Training group (*n* = 208)			Testing group (*n* = 89)			*p* value
	FH (*n* = 84)	UFH (*n* = 124)	*p* value	FH (*n* = 36)	UFH (*n* = 53)	*p* value	
Age (months)	13.5 (3.0, 36.75)	36.50 (20.25, 51.00)	< 0.001	11.5 (4.5, 39.0)	46.0 (26.5, 71.5)	< 0.001	0.205
Gender			0.682			0.857	0.830
Male	45 (53.6%)	70 (56.5%)		19 (52.8%)	29 (54.7%)		
Female	39 (46.4%)	54 (43.5%)		17 (47.2%)	24 (45.3%)		
Location			NA			NA	NA
Neck	1 (1.2%)	0 (0.0%)		0 (0.0%)	1 (1.9%)		
Neck-thorax	1 (1.2%)	0 (0.0%)		1 (2.8%)	0 (0.0%)		
Thorax	9 (10.7%)	12 (9.8%)		5 (13.9%)	0 (0.0%)		
Thorax-abdomen	0 (0.0%)	3 (2.4%)		2 (5.5%)	1 (1.9%)		
Abdomen	63 (75.0%)	105 (84.6%)		22 (61.1%)	47 (88.7%)		
Abdomen-pelvis	5 (6.0%)	2 (1.6%)		2 (5.6%)	4 (7.5%)		
Pelvis	5 (5.9%)	2 (1.6%)		4 (11.1%)	0 (0.0%)		
INRG stage			< 0.001			< 0.001	0.911
L1	17 (20.3%)	14 (11.3%)		8 (22.2%)	3 (5.7%)		
L2	50 (59.5%)	30 (24.2%)		21 (58.3%)	12 (22.6%)		
M	10 (11.9%)	79 (63.7%)		5 (13.9%)	36 (67.9%)		
MS	7 (88.3%)	1 (0.8%)		2 (5.6%)	2 (3.8%)		
Histological subtype			< 0.001			< 0.001	0.522
Poorly differentiated neuroblastoma	49 (58.3%)	96 (77.4%)		24 (66.7%)	36 (67.9%)		
Undifferentiated neuroblastoma	0 (0.0%)	11 (8.9%)		0 (0.0%)	9 (17.0%)		
Differentiated neuroblastoma	2 (2.4%)	1 (0.8%)		1 (2.8%)	1 (1.9%)		
Intermixed ganglioneuroblastoma	29 (34.5%)	0 (0.0%)		11 (30.6%)	0 (0.0%)		
Nodular ganglioneuroblastoma	4 (4.8%)	16 (12.9%)		0 (0.0%)	7 (13.2%)		

*FH* favorable histology, *UFH* unfavorable histology, *NA* not applicable, *INRG* international neuroblastoma risk group

### Feature dimensionality reduction

**Fig. 4:**
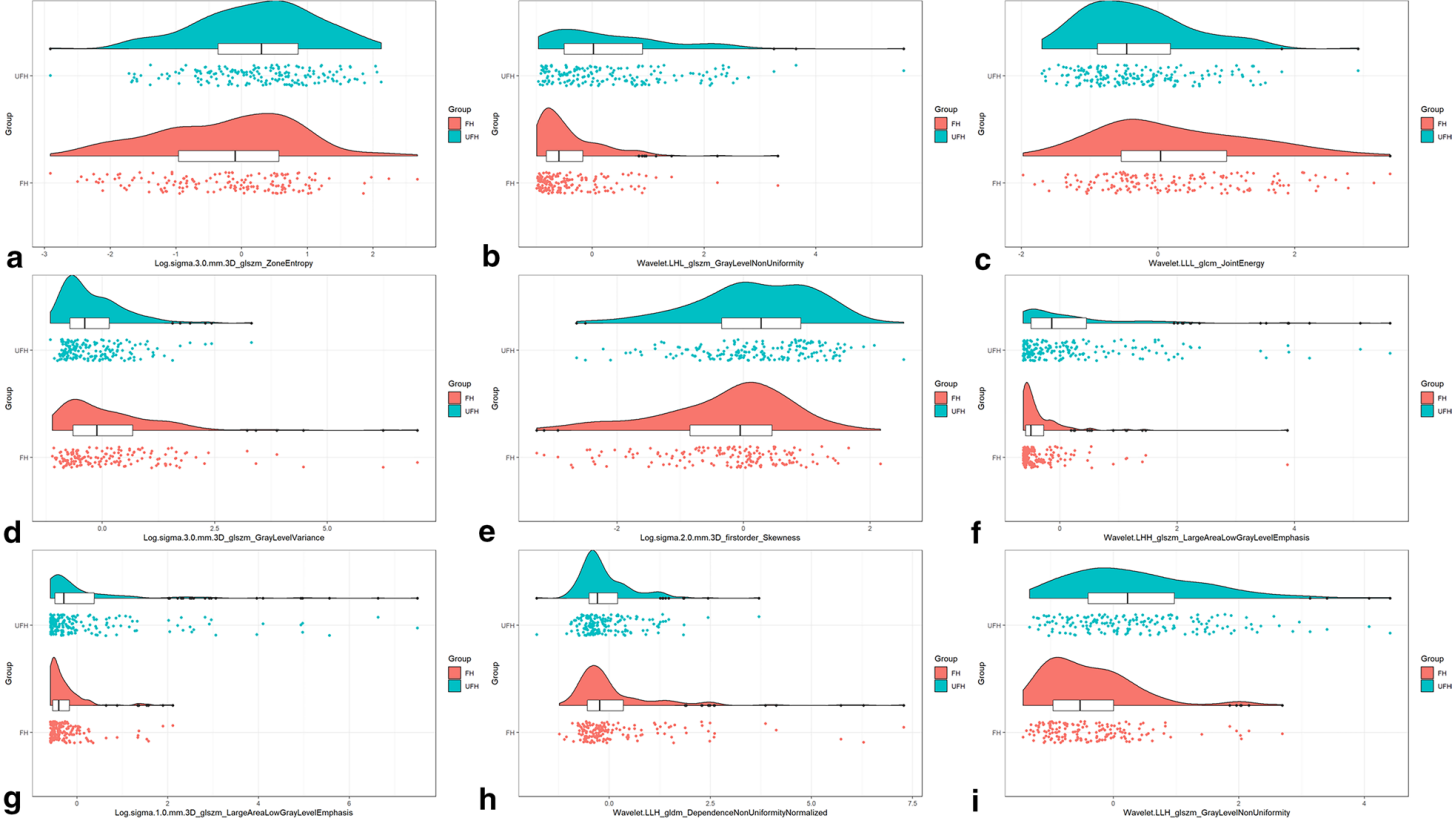
Raincloud plots of radiomics features between favorable histology (FH) and unfavorable histology (UFH) in the entire dataset

Out of the 40 selected cases, there were 25 males and 15 females, with a median age of 14.0 (4.25, 47.75) months and a range of 0.09–114 months, including 20 FH cases and 20 UFH cases. The ICC of radiomics features extracted from twice tumor segmentation had a range from 0.02 to 0.99, with an average ICC of 0.90 and a standard deviation of 0.15. Out of the 168 radiomics features, the ICC of those was found to be less than 0.80. After the removal of features with a PCC greater than 0.99, 593 radiomics features were left. Subsequently, a total of 17 features were retained after LASSO selection. These retained radiomics features are shown in Table 3, with their corresponding distribution in the entire dataset displayed in Fig. 4. The raincloud plots of the remaining features can be seen in Additional file 1: Fig. S2.

**Table 3:** The final radiomics features selected by a least absolute shrinkage and selection operator algorithm

Filter	Type	Subtype
Log.sigma.3.0.mm.3D	glszm	ZoneEntropy
Wavelet.LHH	glszm	LargeAreaLowGrayLevelEmphasis
Log.sigma.1.0.mm.3D	glszm	LargeAreaLowGrayLevelEmphasis
Wavelet.LLH	glszm	GrayLevelNonUniformity
Wavelet.HHL	glcm	Imc2
Log.sigma.2.0.mm.3D	Firstorder	Skewness
Wavelet.HLH	Firstorder	RootMeanSquared
Original	Shape	Maximum2DDiameterRow
Wavelet.LLL	glszm	ZoneEntropy
Original	Shape	Sphericity
Wavelet.HLH	Firstorder	Kurtosis
Wavelet.LLH	Firstorder	Median
Wavelet.LLH	gldm	DependenceNonUniformityNormalized
Wavelet.LHL	glszm	SizeZoneNonUniformityNormalized
Log.sigma.3.0.mm.3D	glszm	GrayLevelVariance
Wavelet.LLL	glcm	JointEnergy
Wavelet.LHL	glszm	GrayLevelNonUniformity

*Log* Laplacian of Gaussian, *glszm* gray level size zone matrix, *glcm* gray level co-occurrence matrix, *gldm* gray level dependence matrix

### Diagnostic performance of the radiomics model

**Fig. 5:**
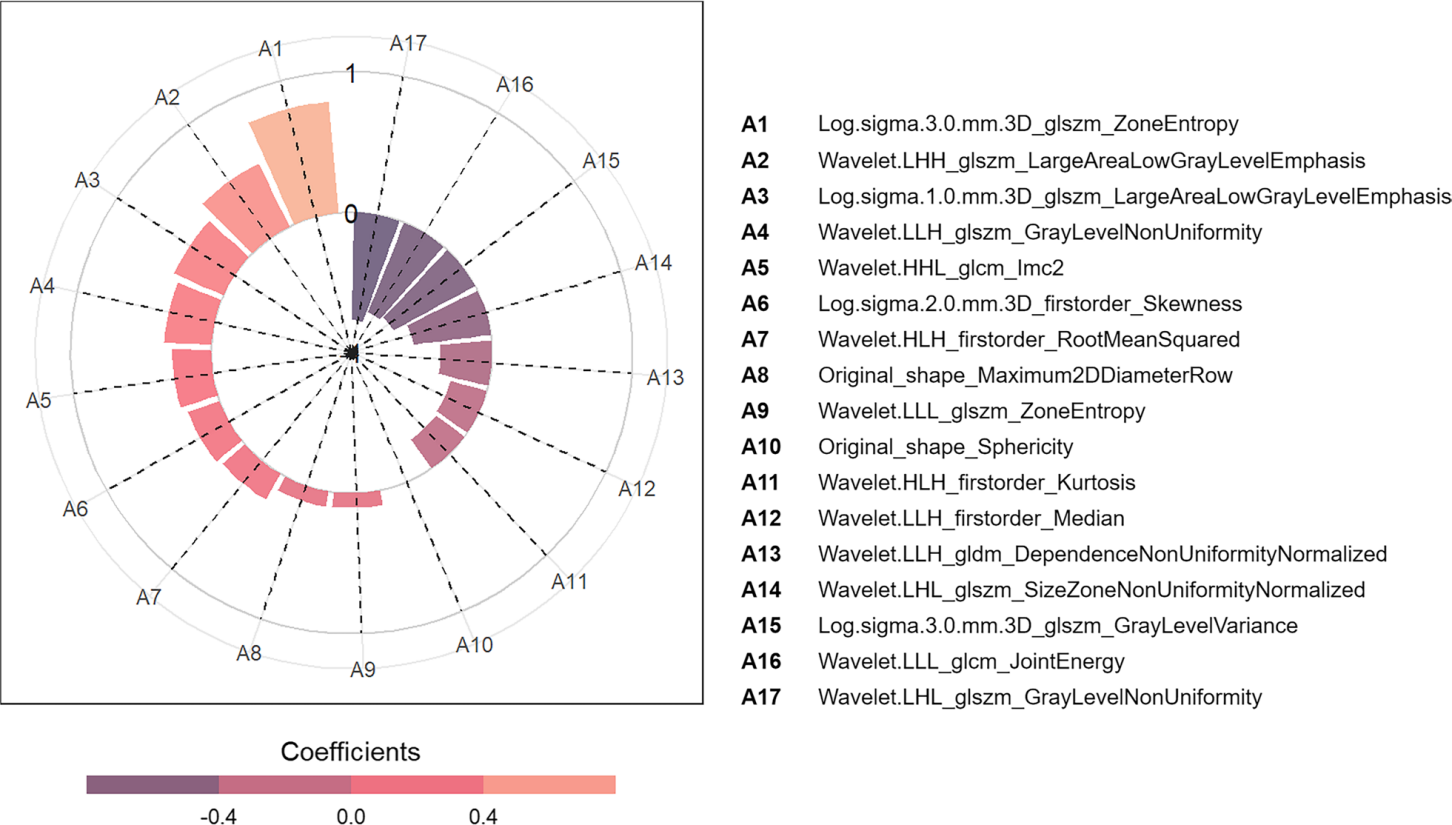
Coefficients of the final radiomics features incorporated in the logistic regression model

**Fig. 6:**
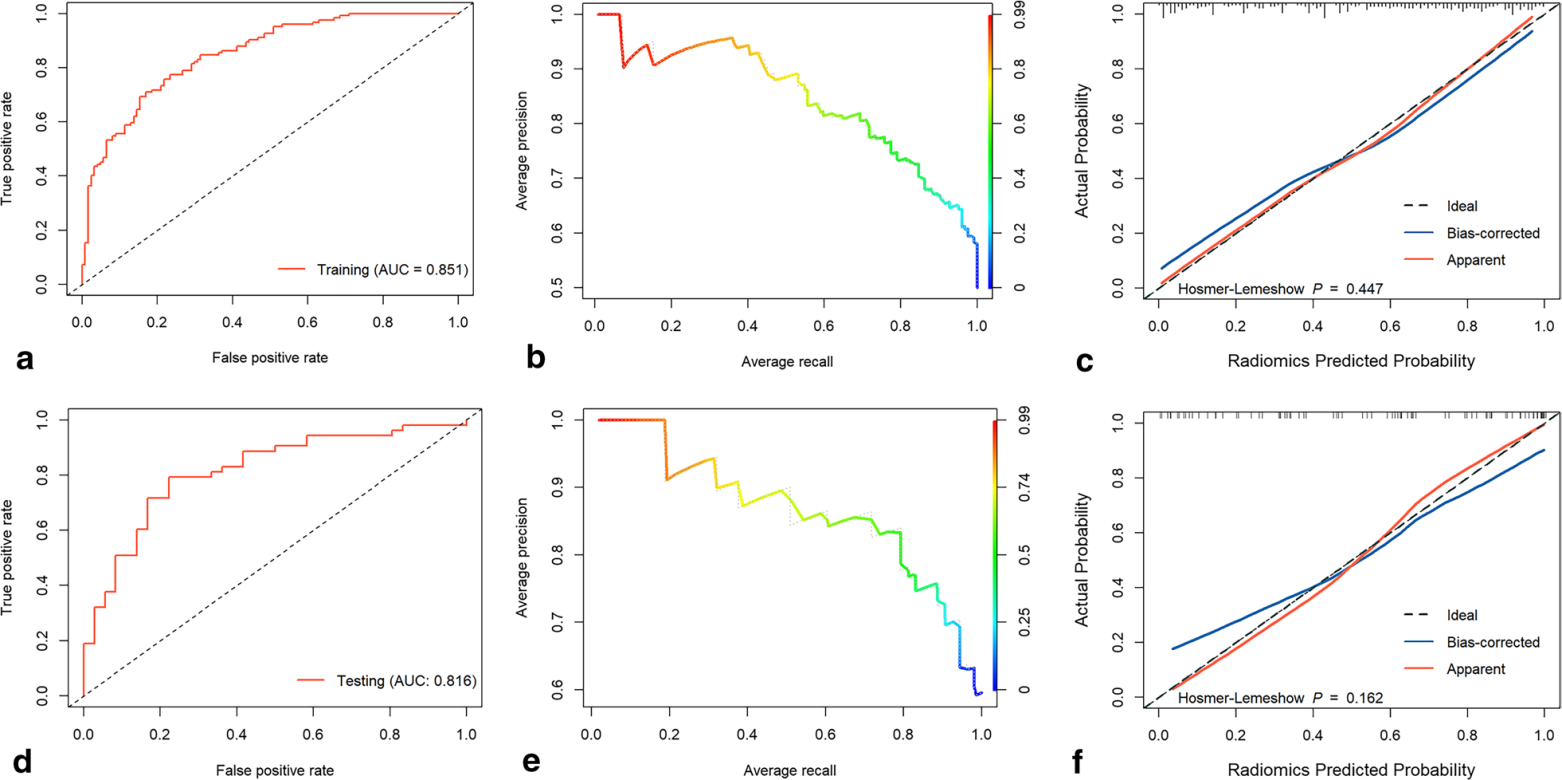
Diagnostic performance of the radiomics model. Figures **a**–**c** show the receiver operating characteristic curve, precision-recall curve, and calibration curve in the training group. Figures **d**–**f** show the receiver operating characteristic curve, precision-recall curve, and calibration curve in the testing group. Hosmer–Lemeshow goodness-of-fit test confirmed that radiomics model fitted well in the training group (*p* = 0.447) and testing group (*p* = 0.162)

**Fig. 7:**
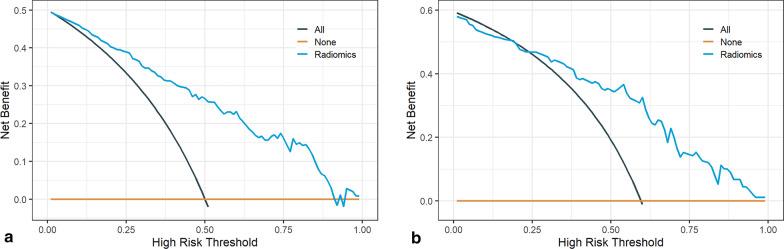
Decision curves of the radiomics model in the training group (**a**) and testing group (**b**). The blue curve indicated the net benefit of the radiomics model at different high-risk threshold

The coefficients of the radiomics features used to construct logistic regression model are presented in Fig. 5. The results of the radiomics model in the training group showed an AUC of 0.851 (95% CI 0.805–0.897), accuracy of 0.770, sensitivity of 0.694, and specificity of 0.847. The evaluation of the radiomics model in the testing group revealed an AUC of 0.816 (95% CI 0.725–0.906), accuracy of 0.787, sensitivity of 0.793, and specificity of 0.778. The details of the evaluation indicators of the radiomics model in both the training and testing groups can be found in Table 4. Hosmer–Lemeshow goodness-of-fit test indicated that radiomics model fitted well in both the training and testing groups (both *p* > 0.05). Figure 6 demonstrates the ROC curves, PR curves and calibration curves of radiomics model in the training and testing groups. Decision curve analysis indicated that radiomics model performed well at different high-risk thresholds in the training and testing groups (Fig. 7).

**Table 4:** Diagnostic performance of the radiomics model in the training and testing groups

Radiomics model	AUC	95%CI	Accuracy	Sensitivity	Specificity	PPV	NPV
Training group	0.851	0.805–0.897	0.770	0.694	0.847	0.819	0.734
Testing group	0.816	0.725–0.906	0.787	0.793	0.778	0.840	0.718

*AUC* area under the curve, *CI* confidence interval, *PPV* positive prediction value, *NPV* negative prediction value

## Discussion

Our study utilized radiomics analysis based on contrast-enhanced CT images to predict INPC subgroups of neuroblastoma. The logistic regression model built on the radiomics features of contrast-enhanced CT images was able to distinguish INPC subgroups of neuroblastoma. The AUC of the radiomics model in the training group was 0.851 (95% CI 0.805–0.897), with an accuracy of 0.770, while the AUC in the testing group was 0.816 (95% CI 0.725–0.906), with an accuracy of 0.787. These results demonstrate that the radiomics model is an effective tool for discriminating INPC subgroups of neuroblastoma. Additionally, the decision curve analysis revealed that the radiomics model could provide clinical benefits.

In the field of medical imaging, radiomics offers a noninvasive and powerful diagnostic tool to uncover the genetically and pathologically heterogeneous features of neuroblastoma. Wu et al. developed a radiomics model based on CT images to predict MYCN amplification in pediatric neuroblastoma, and the AUC of the model was 0.93 (95% CI 0.87–1.00) in the training group and 0.92 (95% CI 0.80–1.00) in the testing group [21]. In another study with a larger sample size, the radiomics logistic regression model was also successful in predicting MYCN amplification status in pediatric abdominal neuroblastoma [22]. In this study, the efficacy of the radiomics model we established to predict INPC subgroups was lower than that of the radiomics models established in other studies to predict MYCN amplification. This may be due to the fact that MYCN, as a risk factor with independent prognostic ability, has a greater influence on the image features of neuroblastoma. In the radiomics model based on ^18^F-FDG PET/CT images for identifying INPC subgroups established by Qian et al. [18], the AUC in the training cohort and validation cohort was 0.877 and 0.868, respectively. This comparatively lower performance in comparison with other studies implies that INPC subtypes are not easily distinguishable [23, 24].

In this study, the final radiomics features used to identify INPC subtypes included gray-level size zone matrix (GLSZM) (8/17), first-order features (4/17), gray-level co-occurrence matrix (GLCM) (2/17), shape (2/17), and gray-level dependence matrix (GLDM) (1/17). The radiomics model was mainly composed of GLSZM, which is a counting matrix that records the number of zones of adjacent connected voxels with the same discrete gray level and is more effective in characterizing texture consistency [25]. In contrast to the run length matrix and the co-occurrence matrix, GLSZM does not require multiple direction calculations. Nevertheless, in Wu et al.'s study, the gray level run length matrix (GLRLM) was highly effective in predicting MYCN amplification [21]. Although the principle of GLRLM is similar to GLSZM, GLRLM mainly records the run length of connected voxels with the same gray level, thus being less affected by the distribution range of voxel values [26]. In contrast to the radiomics model used to identify high-risk neuroblastoma, shape features were found to be less significant in this study [27]. Nevertheless, the results of this study, along with previous research, suggest that texture features play a crucial role in identifying both high-risk and UFH neuroblastoma.

In the ^18^F-FDG PET/CT imaging radiomics model developed to discriminate INPC subgroups, the radiomics features involved in the model were also mainly composed of GLSZM [18]. This indicates that GLSZM is more effective in capturing the image differences between FH and UFH. In this study, GLSZM was mainly composed of LargeAreaLowGrayLevelEmphasis (2/8), GrayLevelNonUniformity (2/8) and ZoneEntropy (2/8). These GLSZM-related texture features represent the uniformity of zone counting at the gray level of the image, indicating the differences in the texture of the contrast-enhanced CT images between the FH and UFH groups. This may be related to the biological behavior of the tumor, as FH is more common in younger children with spontaneous regression or age-appropriate tumor differentiation or maturation, whereas UFH patients are older and have a more heterogeneous molecular signature [28]. Therefore, more attention should be paid to the correlation between GLSZM and the heterogeneous characteristics of neuroblastoma in future studies.

This study has some limitations. The MKI analysis, which is one of the indicators of the INPC classification system, was based on the count of 5000 tumor cells, which could lead to subjective errors and affect the results of the pathological classification. Additionally, the radiomics analysis within the maximum five slices was not ideal. We opted to segment areas of interest in the five largest slices of each tumor due to the large size of the primary tumor, making it difficult and laborious to delineate the entire tumor region of interest in a large sample study. However, in cases of upfront complete resection, it is thought that the histological analysis was taken from the region of the tumor that was prone to radiomics analysis. For primary lesions that underwent biopsy or incomplete resection, INPC classification could only be done when the material was sufficient, so the maximum five slides were very close to the specimen's origin. Furthermore, the use of CT scanning in pediatric patients is a sensitive issue due to the potential radiation exposure it may cause, so this study was limited to a retrospective design. It would be beneficial to investigate if MRI images could provide more information, and conducting multi-center studies could further validate the applicability of the constructed model.

To sum up, the radiomics model that was developed exhibited a good diagnostic performance in discriminating the INPC subgroups of neuroblastoma and could offer a noninvasive approach to assist in the evaluation of the INPC subgroups of neuroblastoma. In the future, the combination of multimodal imaging with functional imaging could potentially enhance the effectiveness of artificial intelligence in the pathological classification of neuroblastoma.

## Supplementary Information

**Additional file 1. **Supplementary Table and Figures.

## Data Availability

All data generated or analyzed during this study are included in this published article.

## Abbreviations

AUC: Area under the curve
CI: Confidence interval
FH: Favorable histology
GLCM: Gray-level co-occurrence matrix
GLDM: Gray-level dependence matrix
GLSZM: Gray-level size zone matrix
ICC: Intra-class correlation coefficient
INPC: International Neuroblastoma Pathology Classification
INRG: International Neuroblastoma Risk Group
LASSO: Least absolute shrinkage and selection operator
LOG: Laplacian of Gaussian
MKI: Mitosis karyorrhexis index
NPV: Negative prediction value
PCC: Pearson correlation coefficient
PPV: Positive prediction value
PR: Precision-recall
ROC: Receiver operating characteristic
UFH: Unfavorable histology
