# Supplementary material for: Radiomics analysis of contrast-enhanced computed tomography in predicting the International Neuroblastoma Pathology Classification in neuroblastoma
Source: Insights Imaging. 2023 Jun 14;14:106. doi: 10.1186/s13244-023-01418-5 (PMC10267098; doi:10.1186/s13244-023-01418-5)
Supplement: Supplementary file 1 — Additional file 1. Supplementary Table and Figures. [file 13244_2023_1418_MOESM1_ESM.pdf]

ELECTRONIC SUPPLEMENTARY MATERIAL

Radiomics analysis of contrast-enhanced computed tomography in predicting the International Neuroblastoma Pathology Classification in neuroblastoma

Supplementary Table 1. Categories of the extracted radiomics features.

Category	Features
Shape	Elongation, Flatness, Least Axis Length, Major Axis Length, Maximum 2D Diameter Column, Maximum 2D Diameter Row, Maximum 2D Diameter Slice, Maximum 3D Diameter, Mesh Volume, Minor Axis Length, Sphericity, Surface Area, Surface Volume Ratio, Voxel Volume
Histogram	10 Percentile, 90 Percentile, Energy, Entropy, Interquartile Range, Kurtosis, Maximum, Mean, Mean Absolute Deviation, Median, Minimum, Range, Robust Mean Absolute Deviation, Root Mean Squared, Skewness, Total Energy, Uniformity, Variance
Texture	Gray Level Co-occurrence Matrix (GLCM), Gray Level Run Length Matrix (GLRLM), Gray Level Size Zone Matrix (GLSZM), Gray Level Dependence Matrix (GLDM)
LOG (sigma: 1.0, 2.0, 3.0)	Histogram, GLCM, GLRLM, GLSZM, GLDM
Wavelet (LLL, LLH, LHL, LHH, HLL, HLH, HHL, HHH)	Histogram, GLCM, GLRLM, GLSZM, GLDM

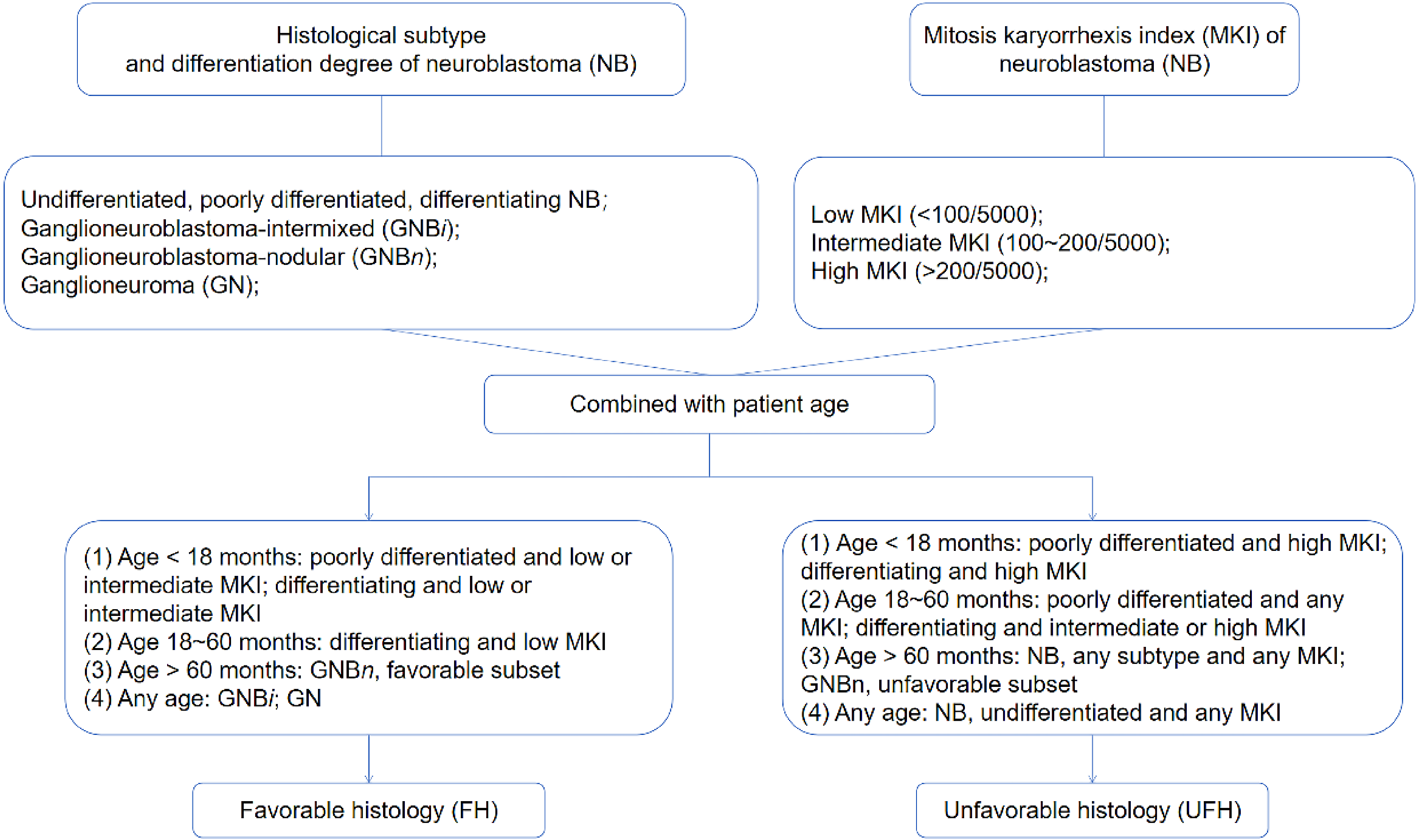

Supplementary Figure 1. Definitions of favorable histology and unfavorable histology in neuroblastoma.

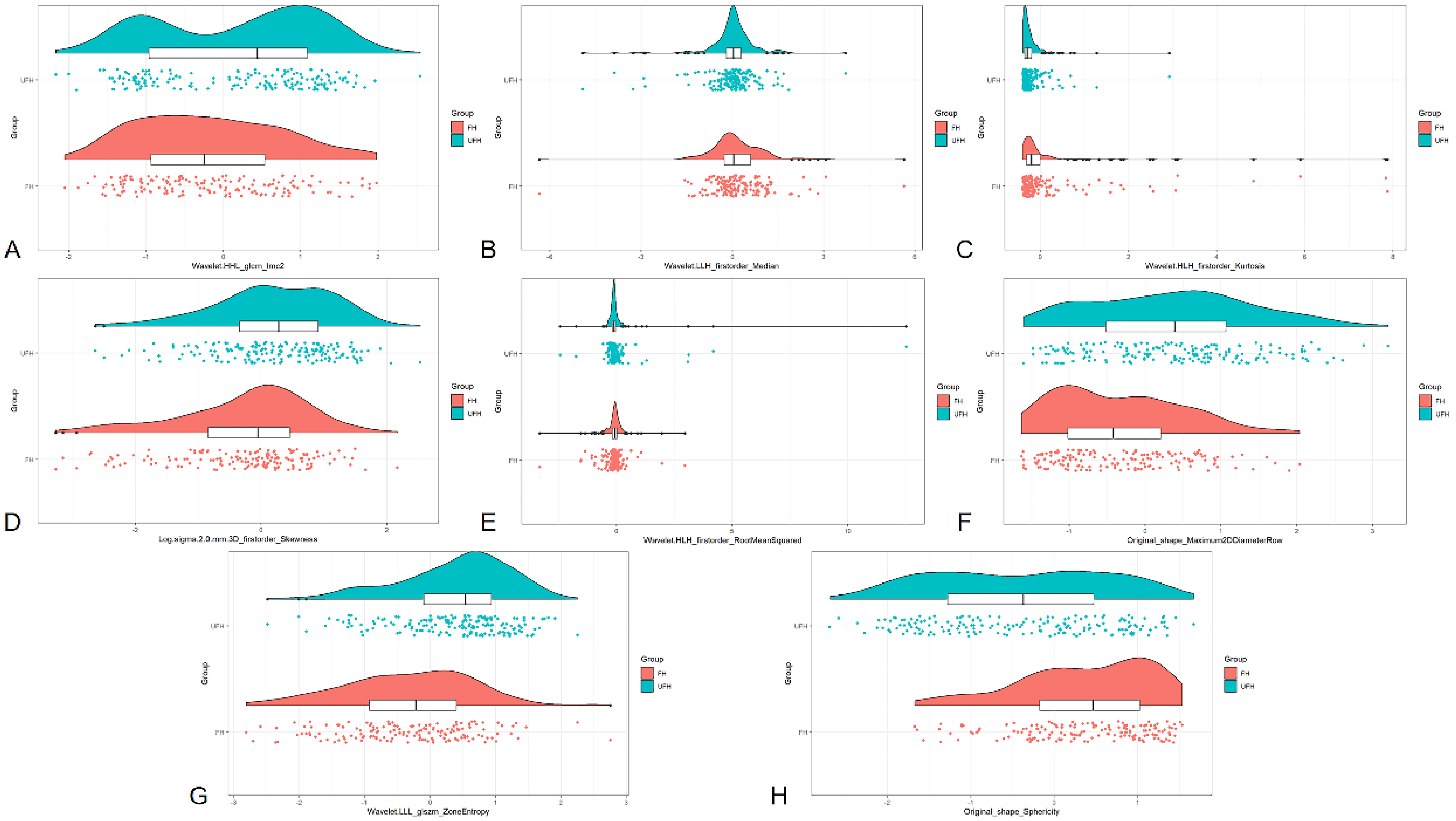

Supplementary Figure 2. Raincloud plots of the remaining radiomics features between favorable histology (FH) and unfavorable histology (UFH) in the entire dataset.
